# Breaking a Guinness World Record on Hand Sanitizing Relay, initiating a call for vital research in overcoming campaign fatigue for hand hygiene

**DOI:** 10.12688/f1000research.5403.2

**Published:** 2015-02-16

**Authors:** Wing Hong Seto, Kwok-Hung Li, Christina Woon Yee Cheung, Patricia Tai Yin Ching, Benjamin J. Cowling

**Affiliations:** 1 Department of Pathology, Hong Kong Baptist Hospital, Kowloon Tong, Hong Kong; 2 Infection Control Unit, Hong Kong Baptist Hospital, Kowloon Tong, Hong Kong; 3 School of Public Health, The University of Hong Kong, Pokfulam, Hong Kong

**Keywords:** Guinness World Record, hand hygeine, Hand Sanitizing, campaign fatigue

## Abstract

Hand hygiene has been shown to be effective in significantly reducing hospital acquired infections for many years. However it is difficult to maintain and enhance compliance with hand hygiene guidelines. In Hong Kong, we previously reported a strategy to counter campaign fatigue from 50%-55% in 2009-11 to 83% in 2012. Here we report a creative activity that we used to sustain and enhance hand hygiene compliance. In May 2014 we broke the first Guinness World Record for a Hand Sanitizing Relay. A total of 277 participants performed hand hygiene before two official and approved witnesses. Following this team-directed strategy, an increase in hand hygiene compliance was identified in June 2014 in two clinical areas with previously poor compliance. The longer term impact of this strategy remains to be determined. More broadly, further research is urgently needed on meeting the challenge of campaign fatigue, and maintaining and enhancing compliance with hand hygiene guidelines.

## Introduction

Hand hygiene (HH) has been shown to be effective in significantly reducing hospital acquired infections for many years ^1^. To ensure that this is practiced all over the world, the World Health Organization (WHO) launched the First Patient Safety Challenge in 2005 ^2^. This is highly successful and as of 24 September 2014 a total of 134 member states of WHO have pledged their support ^3^. The WHO has also provided guidelines on HH with full details on the proper procedures and also guidance on the proper implementation strategy ^4^.

Although the effects of HH are firmly established, it is not easy to achieve compliance to the HH guideline. This is because all staff in the hospital must practice it consistently before, during and after contact with patients and their immediate surroundings. Fully recognizing that compliance is difficult, the WHO guideline has laid out a clear strategy for implementation which includes five essential steps:

Step 1: Facility preparedness including resources and formulating a plan

Step 2: Baseline evaluation – establishing the current situation

Step 3: Implementation – introducing the improvement activities

Step 4: Follow-up evaluation – evaluating the implementation impact

Step 5: Further planning and review cycle.

It should be noted that the WHO also recommends that a plan be developed for the review cycle that is ongoing for a minimum of five years.

The WHO has formulated a Multimodal Hand Hygiene Improvement Strategy (MHHIS) for promoting the HH practice. This strategy is not limited to a single promotional component such a posters and banners. Rather the WHO endorses a myriad of coordinated actions including system change to ensure that alcohol-based handrub and handwashing facilities are in place, continuous training and education programs, HH audits with feedback, reminders in the workplace, and fostering institutional safety climate in the hospital.

## Systematic reviews on efficacy of HH campaigns

Three reviews on HH compliance have been reported in the literature. One review was conducted before 2010 which included all studies in English from a market economy with proper description of results ^5^. A total of 96 studies were included and the results were rather variable but the authors concluded that noncompliance with hand hygiene guidelines is a universal problem and that this can be present even when all the basic requirements for implementation as recommended above were in place. It is stressed that to develop successful interventions, more research on behavioral determinants is needed ^6^. The second is a Cochrane review with updates searches up till November 2009 and was published in 2011 ^7^. The standards for selection were much more stringent and only studies with controls or interrupted time series with explicit entry and quality criteria were considered and finally only four studies were included. Two studies had data on HH compliance and only one showed improvement from the campaign. The other two reported improvement based in increase in usage of hand disinfectants. The authors of the review concluded that soundly designed studies are still required with at least 12 months of follow up. This is because the short duration of the included studies did not permit assessment of the long term impact of the campaigns.

Another review by Huis *et al.* reported in 2012 was conducted but with a focus on classifying the improvement activities based on their determinants of behavior change ^8^. It was stated in the review that the criteria for inclusion were not as stringent as Gould *et al.* ^7^ but rather the standards of Anderson and Sharpe were used ^9^. Nevertheless the papers included must have clearly described their intervention methods for these to be evaluated and finally 41 studies were included. The review concluded that the strategies are rather limited and “we should be more creative in the application of alternative activities”. The authors encouraged further research particularly on group-directed and team-directed strategies in addition to strategies focusing on the individual or organization.

## Recent studies on the long term impact of HH campaigns

As stated in the review by Erasmus *et al.* ^5^, there is a lack of data showing the long term impact of HH campaigns. There are however two studies published in 2014 with data on the impact beyond two years. The first by Myer *et al.* ^10^ reported a significant increase of HH compliance from 22%–40% in 2000 to 65%–81% in 2003–2006. Data for compliance of the different categories of healthcare workers from 2003 to 2006 were also provided and there were no increases recorded during these three years. The compliance data in 2003 and 2006 were the following: physicians (63%; 60%), Nurses (72%; 75%) and other ancillary (65%; 63%). In another study, Biswal *et al.* reported an increase in HH compliance from 28% to 43% after six months ^11^. However an audit conducted two years later shows that compliance had fallen to 36%. These two studies indicate that it is not easy to further enhance compliance in the long term.

## Meeting the challenge of campaign fatigue

The WHO guidelines can be extremely helpful but with the passage of time, maintaining compliance can indeed be problematic. We were the first to report a paper on campaign fatigue in HH, in a hospital in which all components of the WHO MHHIS were utilized ^12^. The compliance to HH successfully increased from 41% to 58% in 2008. However in spite of adding the various components of the MHHIS as recommended, compliance remained at 53% in 2009, 55% in 2010 and 50% in 2011. Compliance that remains static despite active promotional activities is evidence of campaign fatigue. This is similar to advertisement fatigue, defined as the lack of effects on users resulting from frequent and repeated exposures ^13^.

We previously reported a technique to overcome campaign fatigue utilizing the help of frontline link nurses ^12^. This was successful in bringing the HH compliance rate from 50% in 2011 to 83% in 2012. The improvement was noted in all the 19 clinical areas except for two where together there was a decline in compliance from 42% to 10%.

As recommended by Huis *et al.* ^8^ it is important to explore creative alternative activities to promote HH especially for a program that is ongoing for an extended period. Breaking a world record is an exciting endeavor and perhaps this can be one way to meet the challenge but it must be formatted in a manner that will enhance compliance with HH. Breaking the world record for HH had been done before but the record was to be the largest group of participants to do hand washing. It was first started in Bangladesh in 2008, and the latest record was organized by WHO Pan American Health Organization with a total of 740,870 participants in 2011 ^14^. In this world record, the emphasis was on the total number of participants and not on the proper hand hygiene technique and thus there was no attempt to demonstrate any increase in compliance.

**Figure 1.**
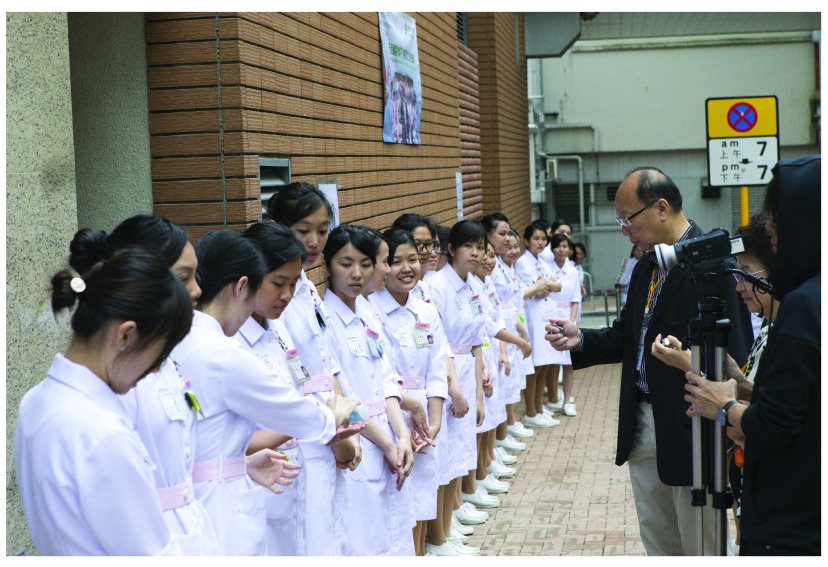
Row of nurses in the relay before the two official validating witnesses, with their stop-watches and the video camera recording each individual performance in the relay.

**Figure 2.**
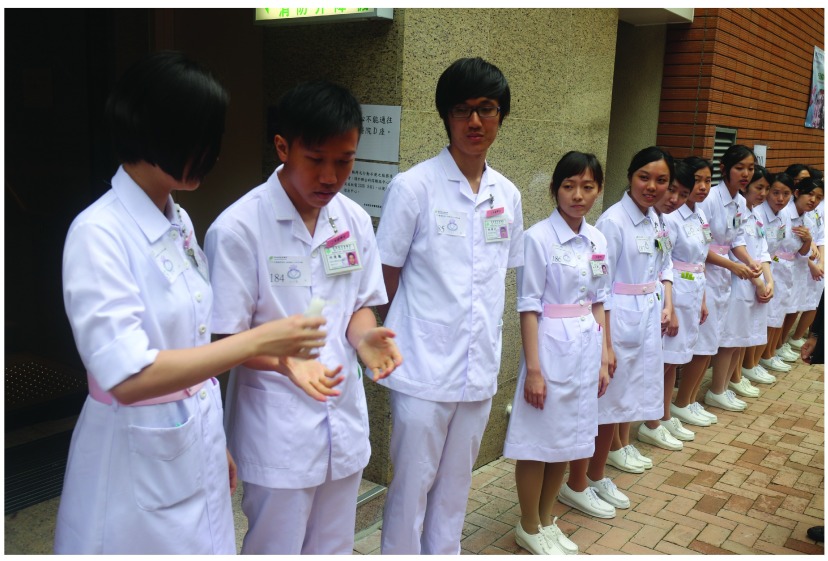
Row of nurses in the relay, the first nurse has just performed hand hygiene correctly and is pouring the alcohol hand rub for the next person.

**Figure 3.**
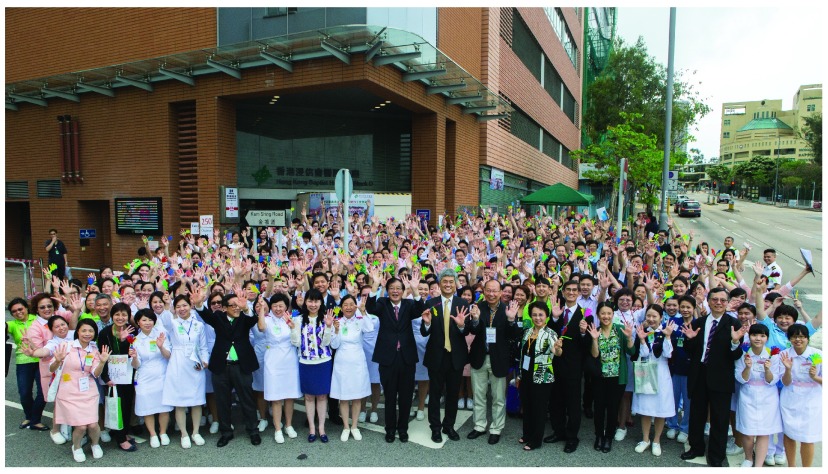
Celebratory photograph after completion of the Hand Sanitizing Relay world record at Hong Kong Baptist Hospital.

In the recent breaking of the first Hand Sanitizing Relay in May 2014, the concept is different. Every individual participant must perform HH before two official and approved witnesses who timed each person using a stopwatch, and recorded by video as required ( Figure 1). It is done in a relay and the participant must perform HH correctly before passing on the alcohol hand rub to the next person ( Figure 2). The entire process was validated by Guinness World Records. In the breaking of this record, a total of 277 participants were involved in the relay ( Figure 3). What is pertinent is that the compliance of the two clinical areas showing a decline in compliance mentioned above increased to 95% in June 2014. It seems that not all health care workers will respond similarly to a promotional campaign and other high impact activities are needed to enthuse the relatively slow movers. Furthermore, Huis *et al.* ^8^ also stressed the need for team-directed strategies and the relay format in this record fits this criterion precisely.

One limitation of our assessment is the observational before-after design, which limits the strength of evidence on its impact. Data on HH compliance in the medium to long term would permit estimation of the rate of decay in compliance, if any.

In conclusion, the essential point to stress in this report is that for health promotional campaigns that are ongoing, it is important to have further research on meeting the challenge of campaign fatigue. This is especially relevant because the HH campaign of WHO is approaching its ten year anniversary and campaign fatigue must certainly be emerging all over the world. Presently the data on this vital subject is meager and indeed more work is needed. The hospital is recognized as one of the most complex organizations in existence. Understanding campaign fatigue is essential not only for HH but also for in the promotion of appropriate healthcare in general.
