# Supplementary material for: Breaking a Guinness World Record on Hand Sanitizing Relay, initiating a call for vital research in overcoming campaign fatigue for hand hygiene
Source: F1000Res. 2015 Feb 16;3:234. Originally published 2014 Oct 3. [Version 2] doi: 10.12688/f1000research.5403.2 (PMC4329673; doi:10.12688/f1000research.5403.2)
Supplement: Peer review history

# Peer review: Referee response for version 1

**DOI:** 10.5256/f1000research.5771.r7083

**Published:** 2015-02-10

**Authors:** Alison Holmes

**Affiliations:** 1 Department of Infectious Diseases, Imperial College London, London, UK

The authors of this study should be praised for approaching the matter of behavioural fatigue from such a refreshing angle. As such, their experience was an innovative activity.

Technically, the paper is well written, although I feel that perhaps the initial sections focusing on evidence regarding hand hygiene campaigns was too long, considering the overall extension of the paper. Likewise, the authors may have been able to benefit related from including some existing evidence from other fields related to overcoming behavioural fatigue.

Whilst the authors indicate that their intervention managed to increase compliance in 2 areas from 10% in 2012 (mentioned in " *...improvement was noted in all the 19 clinical areas except for two where together there was a decline in compliance from 42% to 10%*") to 95% in June 2014, it would have been extremely useful to see the decay rate of such boost (I accept that the authors highlight as a limitation of their paper the need to see the longer term impact of their intervention, but I feel that as the problem they tried to solve was related to compliance fatigue, we would have benefited from examining how well their initiative fared).

But these suggestions should not impact on the merit of the paper, which is showcasing an innovative approach focused on maintaining engagement with desired hand hygiene behaviours.

I have read this submission. I believe that I have an appropriate level of expertise to confirm that it is of an acceptable scientific standard.

# Response

**Published:** 2015-02-11

**Authors:** Ben Cowling

**Affiliations:** Hong Kong University, Hong Kong

Thank you for these comments which we greatly appreciate. We will look into the literature on overcoming campaign fatigue in other fields. We agree that examining the medium to longer term compliance is important and we are continuing to monitor HH compliance in this hospital.

# Peer review: Referee response for version 1

**DOI:** 10.5256/f1000research.5771.r7084

**Published:** 2015-01-19

**Authors:** Vicki Erasmus

**Affiliations:** 1 Department of Public Health, Erasmus MC University Medical Center Rotterdam, Rotterdam, Netherlands

This is an interesting intervention study with a good twist on keeping hand hygiene interesting for healthcare professionals. The intervention can also inspire other hospitals to undertake quality improvement which is excellent! In general the paper is well written with a good literature base, and great effort were undertaken to ensure reliability during the record breaking attempt, including two witnesses. I have two reservations though, and hope the authors could add something on these points.

1. The authors report an increased compliance from 50->83%, although it in unclear how or where this was measured. Was this data collected after the intervention (how long after?) in hospital wards? More details on this could help place the results in perspective and help other professi9onal undertake similar efforts.
2. I miss a discussion of the project. By listing some of their limitations others can be helped in further improving similar activities.

I have read this submission. I believe that I have an appropriate level of expertise to confirm that it is of an acceptable scientific standard, however I have significant reservations, as outlined above.

# Response

**Published:** 2015-02-11

**Authors:** Ben Cowling

**Affiliations:** Hong Kong University, Hong Kong

Thank you for these comments which we greatly appreciate. We have made some minor revisions to our article to address the points made by the reviewer. Specifically, we have now clarified the dates of the Guinness World Record activity and the dates of subsequent assessment of HH compliance, in May 2014 and June 2014 respectively. We have also added a brief discussion of the project and the limitations, which will help others planning similar activities.

# Peer review: Referee response for version 1

**DOI:** 10.5256/f1000research.5771.r6323

**Published:** 2014-11-06

**Authors:** Mary-Louise McLaws

**Affiliations:** 1 School of Public Health and Community Medicine, University of New South Wales, Sydney, NSW, Australia

This manuscript reports on a unique project aimed at refreshing the support for a WHO global hand hygiene compliance program in healthcare workers from a tertiary teaching hospital in Hong Kong. The manuscript is well written and has succinctly reported the literature to highlight the lack of research into methods to amelioration fatigue. The only comment I have to improve on this excellent manuscript is to include a reference about the need for behavioural research (Page 2, paragraph 6),  after “....behavioural determinants is needed” e.g. by Whitby M *et al.* (2007) *.*

I have read this submission. I believe that I have an appropriate level of expertise to confirm that it is of an acceptable scientific standard.

# Response

**Published:** 2015-02-11

**Authors:** Ben Cowling

**Affiliations:** Hong Kong University, Hong Kong

Thank you for these comments which we appreciate. We have incorporated the reference suggested by the reviewer in our revised manuscript.
